# The impact of glucocorticoid receptor transactivation on context-dependent cell migration dynamics

**DOI:** 10.1038/s41598-025-88666-1

**Published:** 2025-02-04

**Authors:** Szonja Polett Pósa, Éva Saskői, Lili Bársony, Lőrinc Pongor, Fanni Fekete, János Papp, Anikó Bozsik, Attila Patócs, Henriett Butz

**Affiliations:** 1 Department of Molecular Genetics and the National Tumor Biology Laboratory, National Institute of Oncology, Budapest, Hungary; 2 Cancer Genomics and Epigenetics Core Group, HCEMM, Szeged, Hungary; 3 Department of Oncology Biobank, National Institute of Oncology, Budapest, Hungary; 4 Hungarian Research Network, HUN-REN-OOI-TTK-HCEMM Oncogenomics Research Group, Budapest, Hungary; 5 Department of Laboratory Medicine, Semmelweis University, Budapest, Hungary

**Keywords:** Glucocorticoid, Glucocorticoid receptor, Breast cancer, Migration, Transcriptome, Gene expression, Cancer, Molecular biology, Endocrinology, Molecular medicine, Pathogenesis

## Abstract

The glucocorticoid receptor (GR) plays a significant role in breast cancer cell behaviour, although data on its effects are conflicting. The impact of GR agonist dexamethasone (dex) and antagonist mifepristone (mif) on oestrogen-positive (ER+) and triple-negative (TN) breast cancer cell lines in both 2D and 3D cultures was studied using multiple in vitro functional assays and transcriptome sequencing. GR activation increased cell motility in TN but not in ER + tumour cells, as observed in both collective and single-cell migration tests. Time-lapse analysis showed enhanced motility after 4–6 h in wound healing, despite dex inhibiting migration initially. This inhibition was observed at 2 h in single-cell tracking migration assays. Cell proliferation increased in TN and decreased in ER + cells upon GR activation, reversed by GR antagonist. RNA sequencing revealed dex’s impact on cell adhesion and extracellular matrix signalling in TN cells and on DNA replication in ER + cells. Based on data from 1085 human breast cancer specimens, GR pathway expression correlated with migratory, extracellular matrix, and angiogenesis gene signatures. Additionally, higher expression of GR and increased GR signature were observed in fast-migrating cells compared to slow-migrating ones. Positive correlation between the GR signature and migration signature at the single-cell level indicated an association between GR activity and cell migration. For the first time, we assessed altered time-lapse migration dynamics in TN breast cancer cells, potentially contributing to cancer progression and prognosis, highlighting that the effects of dexamethasone on breast cancer cell migration are influenced by ER status and treatment duration.

**Supplementary Information:**

The online version contains supplementary material available at 10.1038/s41598-025-88666-1.

## Introduction

Breast cancer, with over 2 million new cases annually, is the most common cancer among women worldwide and a leading cause of cancer-related deaths^1^. This neoplasm includes a range of subtypes distinguished by specific histopathologic, molecular and clinical characteristics, highlighting its highly heterogeneous nature. Histopathological classification essentially determines the disease prognosis and indicates the effective therapy^2^. The molecular subtypes of breast cancer, based on immunohistochemical expression, are typically classified into four categories: oestrogen receptor-positive (ER+), progesterone receptor positive (PR+), human epidermal growth factor receptor-positive (HER2+), and triple-negative (TNBC). The latter refers to those with a lack of expression of these receptors and is generally associated with poor prognosis^2^. Chemotherapy administered for breast cancer patients may induce adverse effects (such as anaphylaxis, nausea and vomiting, anorexia, low energy level etc.). Managing these treatment-related side-effects is crucial which involves the routine use of synthetic glucocorticoids (GCs), such as dexamethasone (dex)^3^. Nevertheless, recent reviews have emphasized controversial findings on the effects of GCs in breast cancer therapy, with outcomes dependent on the cancer subtype^4–7^.

Remarkably, in patients with ER + breast cancer, elevated levels of GR expression in tumours were strongly linked to a more favourable outcome, while worse long-term survival and more recurrence are expected in ER- and TN tumours with greater expression of GR^8–10^. Hence, it is of significant clinical importance to gain a deeper understanding of the molecular mechanisms behind the context-dependent actions of GR. These mechanisms govern the role of GCs in cancer cells and the progression of breast cancer. These areas remain incompletely elucidated thus far.

Prior studies also suggest that GCs can inhibit cell proliferation and block oestrogen-induced stimulatory effects in ER + breast cancers in vitro, but a potential concern has been raised regarding their impact on TNBCs, where they were shown to promote cancer growth and induce metastasis both in vitro and in vivo^3–5^.

Although migration has a central role in tumour progression and metastasis development, only scarce literature data have been available about the process of breast cancer cell migration^11,12^. In addition, cell motility has been also scarcely investigated in association with GR action^13,14^.

Our study aimed to investigate the effects of the GR agonist dex on breast cancer cell growth, proliferation, and migration in relation to ER status and treatment duration. We also sought to explore the transcriptomic background of the dex response and the correlation of GR-related gene signatures with tumour progression-associated signalling pathways in clinical breast cancer samples.

Specifically, we examined dexamethasone’s mechanisms in regulating growth, viability, and motility across breast cancer cell lines with ER + and TN receptor profiles in both monolayer (2D) and spheroid (3D) cultures. To investigate the process of cell motility and migration, besides classic wound healing tests, we firstly applied single-cell migration tracking in time-lapse fashion to accurately characterize the effect of dex on migration dynamics. Transcriptome sequencing was also done to analyse the gene expression changes and related signalling pathways involved in the effects of dex in the context of the oestrogen receptor and on breast cancer progression. For validation, gene signatures related to migration, extracellular matrix, and angiogenesis were evaluated in the context of glucocorticoid receptor (GR) in a reanalysis of single cell and bulk transcriptome sequencing data from 1085 human breast cancer samples.

## Results

### Effect of ligand-dependent glucocorticoid receptor activation on 2D and 3D breast cancer cells

**Fig. 1:**
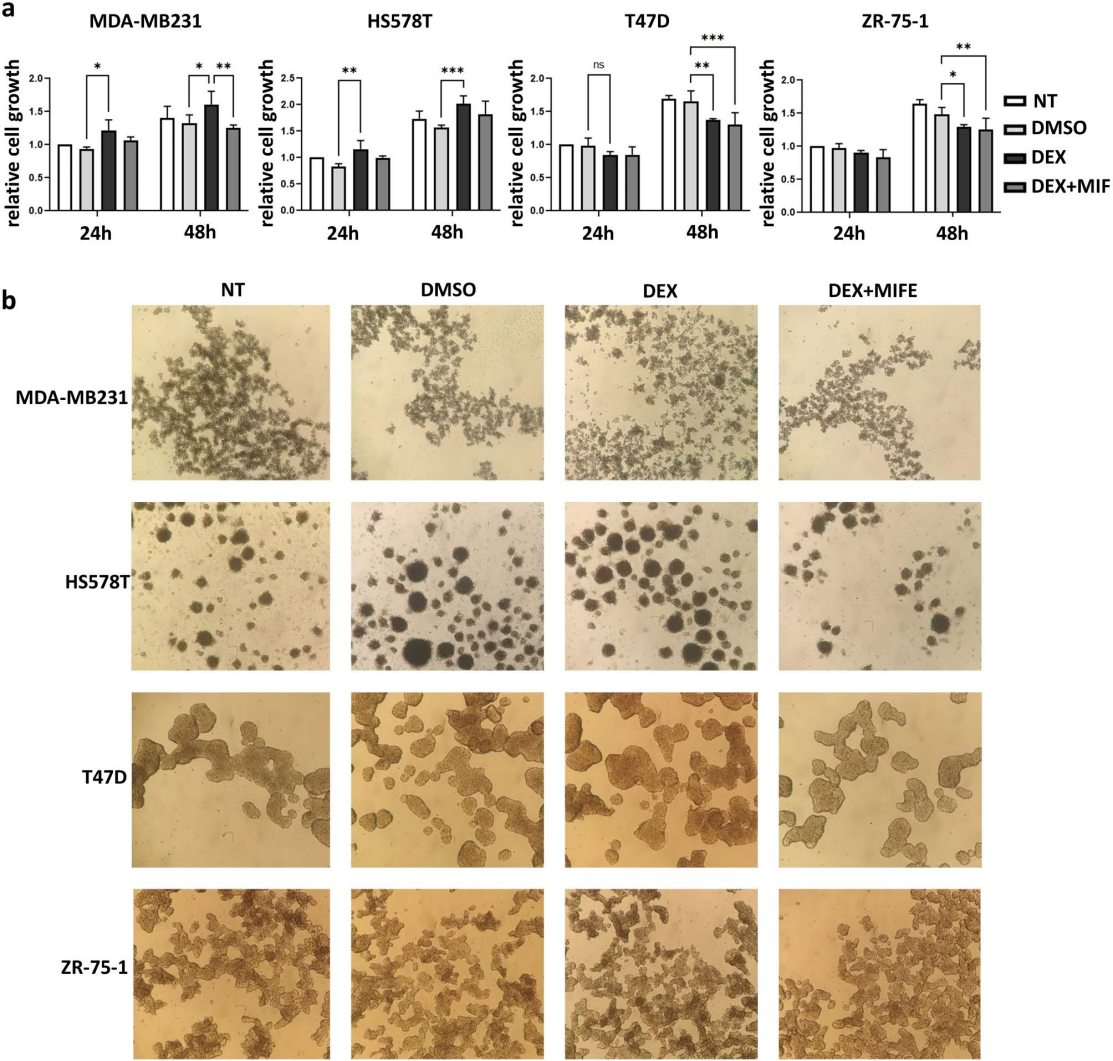
Dexamethasone effect on TN and ER + breast cancer cell growth**. a** Cell proliferation of breast cancer cells upon GR agonist (dexamethasone, dex) and antagonist (mifepristone, mif) treatment. Columns represent the averages of three biological replicates, with error bars indicating standard deviations. Statistical differences were assessed by ANOVA following Dunnett test to compare different groups. *: *p* ≤ 0.05; **: *p* ≤ 0.01; ***: *p* ≤ 0.001. **b** No morphological or cell proliferation change have been observed in 3D spheroid cultures.

To investigate ligand-dependent GR activation, we treated two ER+ (T47D and ZR-75-1) and two ER- (TN) breast cancer cell lines (MDA-MB231 and HS578T) with GR agonist (dexamethasone, dex) with and without GR antagonist (mifepristone, mif). We found that in monolayer cultures dex increased cell proliferation in MDA-MB231 and HS578T that was eliminated by GR antagonist (Fig. 1A). However, in the T47D and ZR-75-1 cell lines, dex decreased cell growth, which was not affected by the combined treatment. We found no morphology changes in 3D spheroid cultures upon dex or combined treatments (Fig. 1B). In terms of cell growth, similar results were observed in 3D spheroids compared to monolayer cultures: proliferation was induced by dexamethasone by 1.45- and 1.32-fold in MDA-MB231 and HS578T cells, while it was decreased by 0.69- and 0.65-fold in T47D and ZR-75-1 (*p* = 0.039). Interestingly, in 3D spheroid cultures, mifepristone was able to counteract the dexamethasone effect in all cell lines irrespective of the presence of ER.

### Altered dexamethasone-induced migration dynamics

**Fig. 2:**
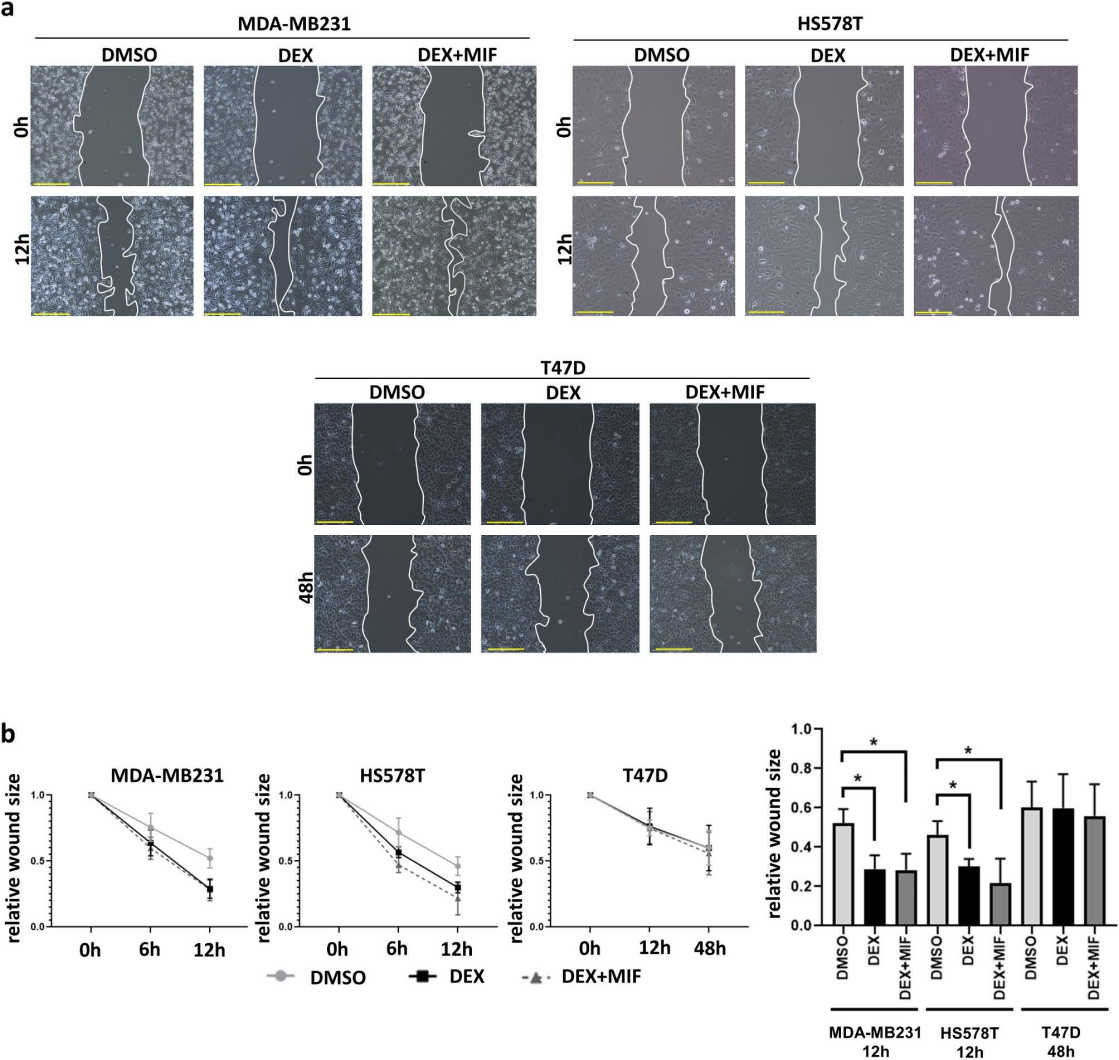
Dexamethasone effect on TN and ER + breast cancer cell migration.** a** Microscopic images of collective cell migration test by wound healing assay upon GR agonist (dexamethasone, dex) and antagonist (mifepristone, mif) treatment in TN (MDA-MB231 and HS578T) and ER+ (T47D) cell lines. **b** Scratch wound closure was faster in triple-negative (TN) MDA-MB231 and HS578T cells upon both dex and mif treatment compared to vehicle control. Data points/columns represent the averages of three biological replicates, with error bars indicating standard deviations. Statistical differences were assessed by ANOVA following Tukey’s test to compare different groups. *: *p* ≤ 0.05.

Wound healing was initially assessed by microphotographic analysis at 0 h, 6 h, and 12 h for MDA-MB231 and HS578T cells, and at 0 h, 12 h, and 48 h for T47D and ZR-75-1 cells considering the different migration rate. The wound healing assay in TN cells indicated that dex increased cell migration, while it did not influence cell motility in ER + cell lines. Combined treatment with mifepristone was not able to antagonize the effect of dex (Fig. 2A-B).

**Fig. 3:**
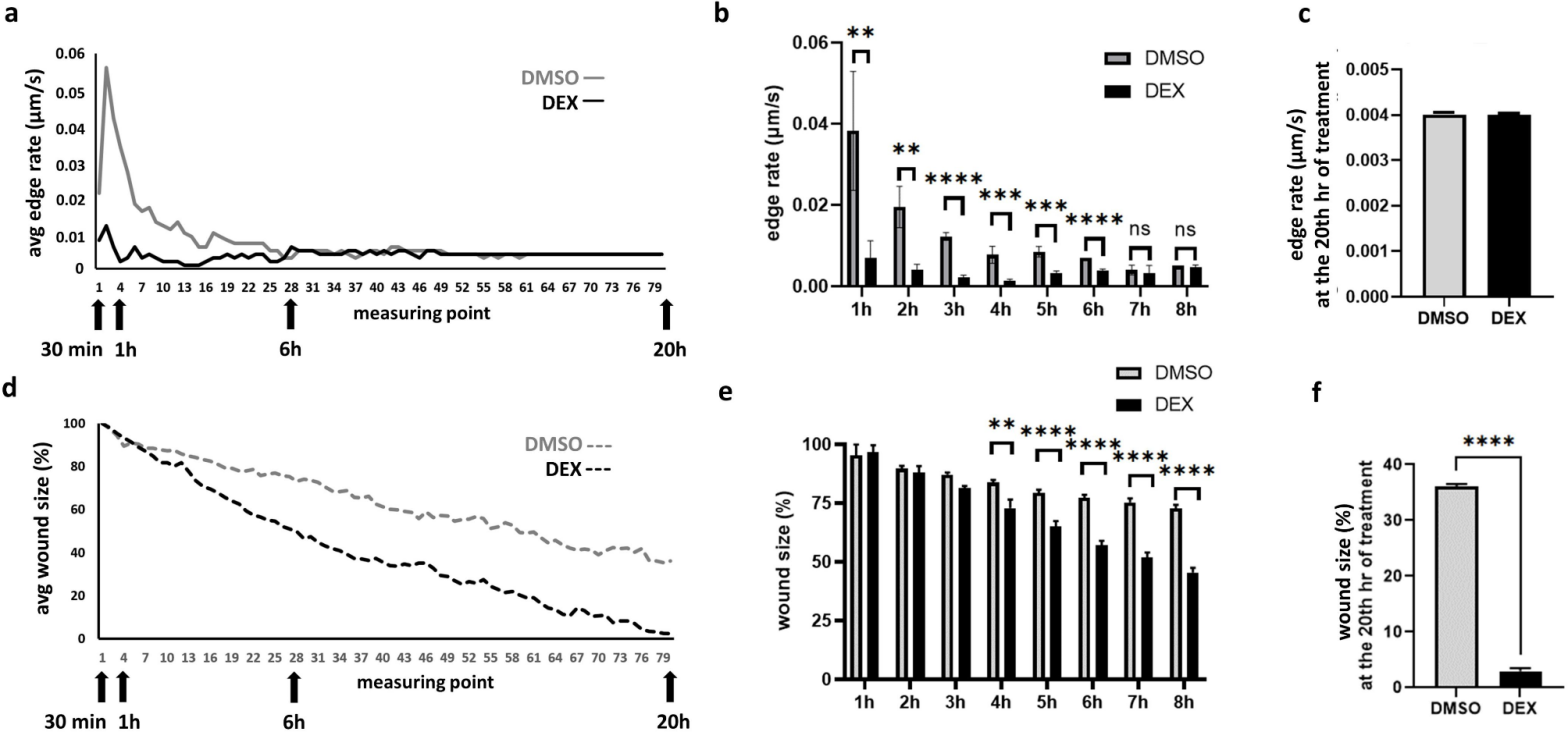
Characteristics of collective migration dynamics. **a** Representative example of dynamics of collective cell migration change upon dex treatment. Dex treatment initially—in the first 6 h—inhibits edge rate compared to the control. **b** Egde rate was higher in the 1st hour of treatment in vehicle (DMSO) controls, while it was balanced after the 6th hour of treatment. **c** Edge rate after 6 h could not be reliably evaluated due to the low rate (0.01 μm/s), which is a limitation of our instrument. However, wound sizes indicated a dex-induced migration-promoting effect compared to the controls: **d** Representative example of wound closure upon dex treatment. **e-f** Wound size significantly reduced in the dex treated condition after the 4-6th hour of treatment compared to controls. Columns represent the averages of four biological replicates, with error bars indicating standard deviations. For statistical comparison of DMSO- and dex-treated conditions, in each time points they were compared using unpaired t-test. ns: *p* > 0.05; *: *p* ≤ 0.05; **: *p* ≤ 0.01; ***: *p* ≤ 0.001; ****: *p* ≤ 0.0001.

**Fig. 4:**
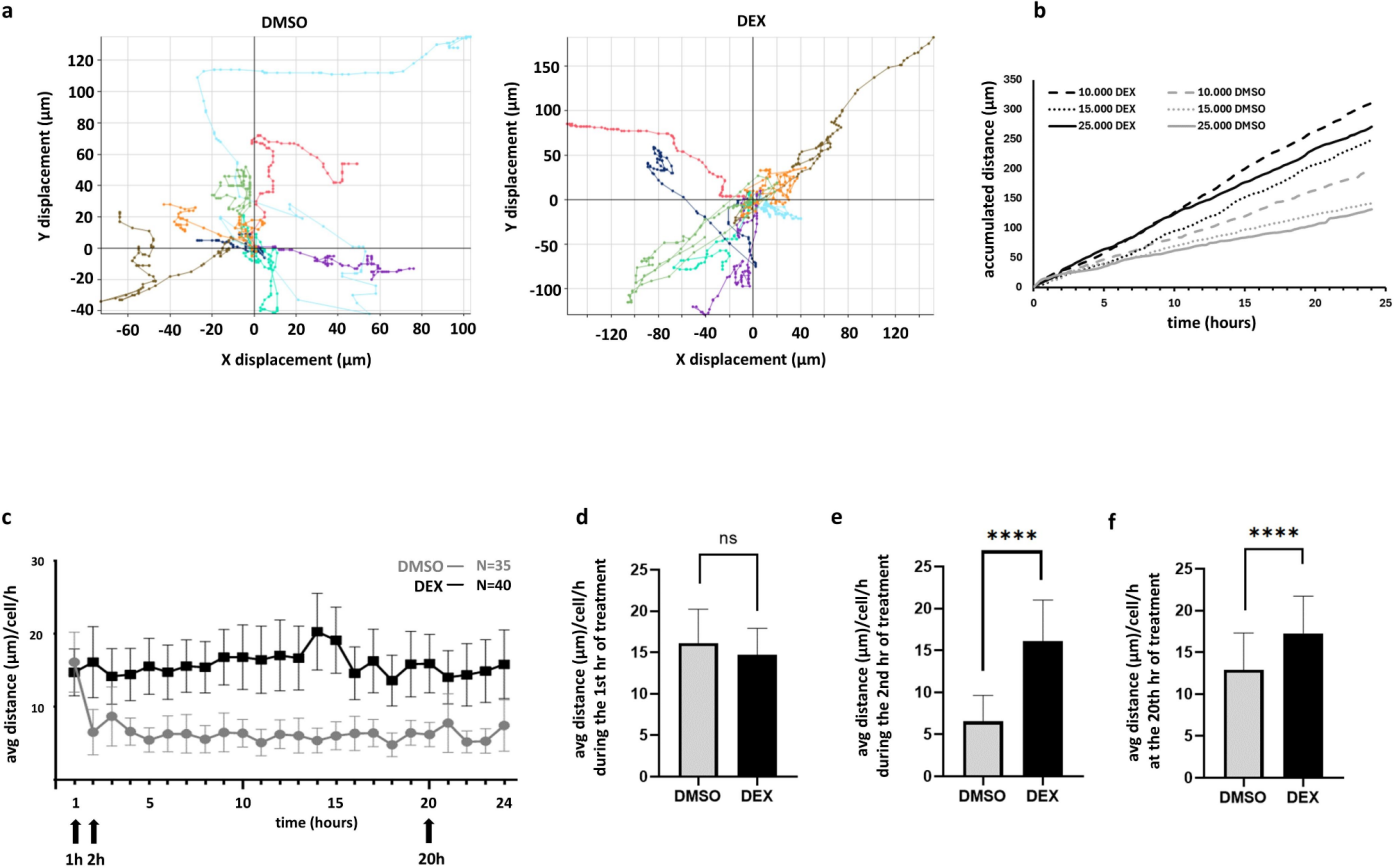
Characteristics of single-cell migration dynamics. **a** Representative image showing single-cell migration distances. Variability in migration distance was observed across individual cells in both dex- and vehicle (DMSO)-treated groups; however, dex treatment increased the accumulated migration distance, as indicated by the differently scaled axes. **b** Accumulated migration distance was influenced by cell seeding density but increased independently of seeding density under dex treatment. **c-f** During the first hour of treatment, vehicle (DMSO)-treated cells showed faster migration compared to later hours, while the migration-promoting effect of dex became evident as early as the second hour. Data in panels **c-f** represent the average migration distances of 35 control (DMSO) and 40 dex-treated cells tracked across three experiments. Statistical comparisons between DMSO and dex treatments at each time point were conducted using an unpaired t-test. ns: *p* > 0.05; ****: *p* ≤ 0.0001.

In independent time-lapse imaging experiments, collective migration dynamics were influenced in a time-dependent manner in TN breast cancer cell lines MDA-MB231 and HS578T. Dex initially decreased edge rate significantly at 60 min, with this difference being eliminated after 6 h (Fig. 3A-C). (Edge rate after 6 h could not be reliably evaluated due to the low rate (0.01 μm/s), which is a limitation of our instrument). This migration-promoting effect manifested as a significant difference in wound size after 4–5 h (Figs. 3D-F and 4A-F).

In single-cell migration experiments, we observed heterogeneity among individual cells (Fig. 4A). Although the number of seeded cells influenced migration (Fig. 4B), independently of this, the effect of dex increased individual cell migration after the 2nd hour of treatment (Fig. 4C-F). Regarding time-lapse individual migration dynamics, we observed a similar pattern as in collectively migrating cells during wound healing assay (Fig. 4C-F). The migration-promoting effect, however, appeared earlier (already after 2 h – Fig. 4C-E) compared to collective migration (4–6 h). Naturally, the overall accumulated distance during the whole 24-hour monitoring of dex treatment exhibited increased values compared to controls.

### Context-dependent GR-mediated gene transactivation in breast cancer cells

**Fig. 5:**
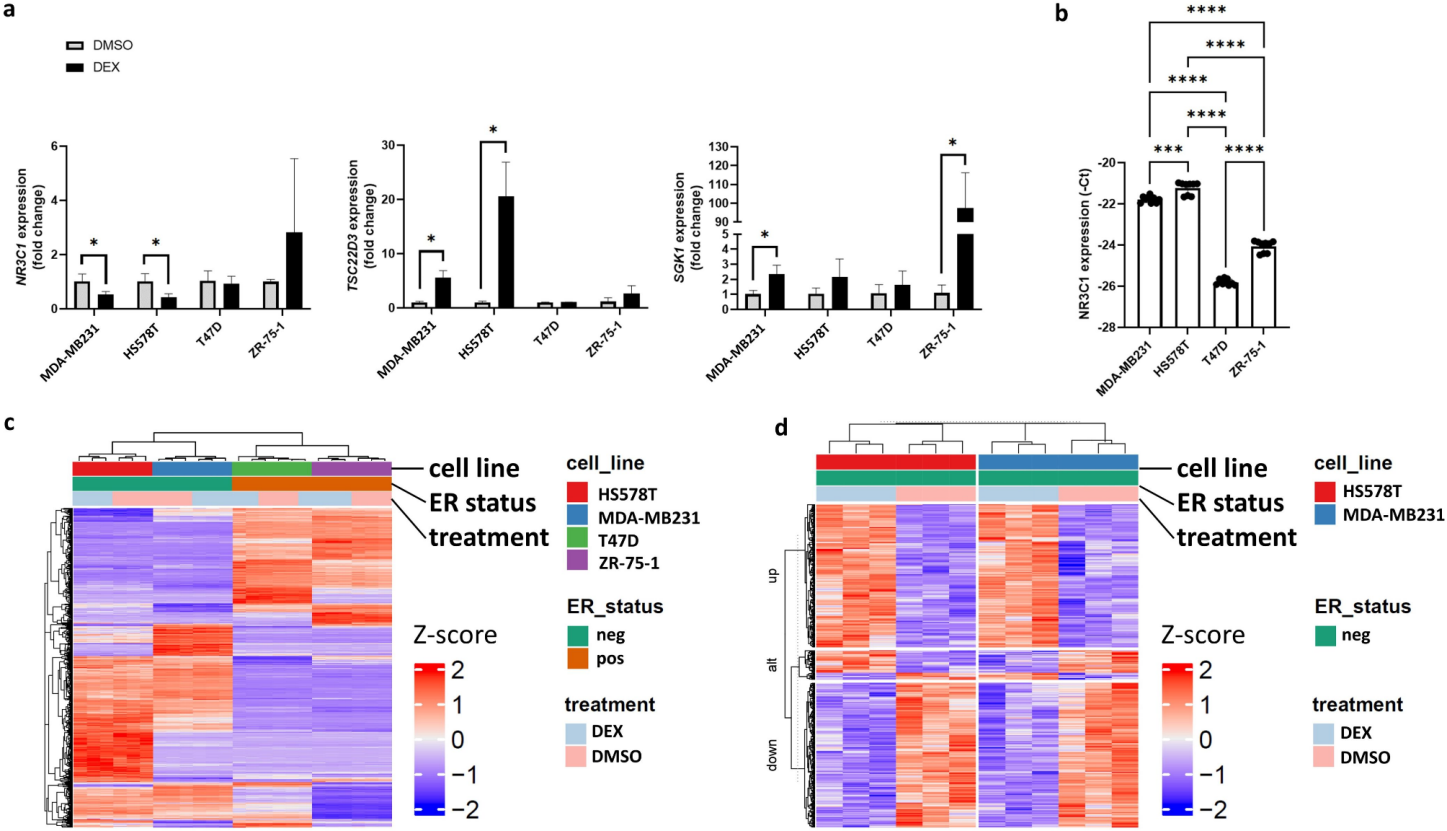
**a** Gene expression changes following dex treatment. Statistical comparisons between DMSO and dex-treated conditions at each time point were performed using an unpaired t-test. **b ***NR3C1* expression levels varied across the four cell lines, with the lowest expression observed in T47D cells. Statistical significance was assessed by ANOVA followed by Tukey’s test for multiple comparisons. ***: *p* ≤ 0.001; ****: *p* ≤ 0.0001. Columns in panels (**a-b**) represent the averages of three biological replicates, with error bars showing standard deviations. **c** Heatmap illustrating the global transcriptomic profile of breast cancer cell lines treated with vehicle control (DMSO) and dex. **d** Heatmap of differentially expressed genes between DMSO- and dex-treated conditions. “up” and “down” indicate genes commonly up- or down-regulated across cell lines, while “alt” marks genes with treatment-specific expression differences among the cell lines.

Since we did not observe a counteracting effect of the GR antagonist on migration during combined dex and mif treatment, our next step was to assess the effect of dex alone on transcriptome changes. First, we validated the changes of GR target genes: Glucocorticoid-induced leucine zipper (GILZ or *TSC22D3*), Serum- and glucocorticoid-inducible kinase 1 (*SGK1*) and Glucocorticoid receptor (*NR3C1*) by qPCR. We found that GR target genes were induced in MDA-MB231, HS578T and ZR-75-1 cell lines while *NR3C1* was downregulated upon dex effect (Fig. 5A). Interestingly, T47D showed poor transcriptomic response based on GR target gene expression. This phenomenon could be due to the low *NR3C1* expression in T47D cells (Fig. 5B). Additionally, by investigating potential genetic alterations in T47D cells compared to the other three cell lines that could explain the difference in glucocorticoid response, a Tier I (pathogenic) variant, *PIK3CA*(NM_006218.4):c.3140 A > G, p.(His1047Arg), was detected in T47D cells which is one of the *NR3C1* downstream molecule. No other potential causal genetic variant was identified (**Suppl. Table 1**).

**Fig. 6:**
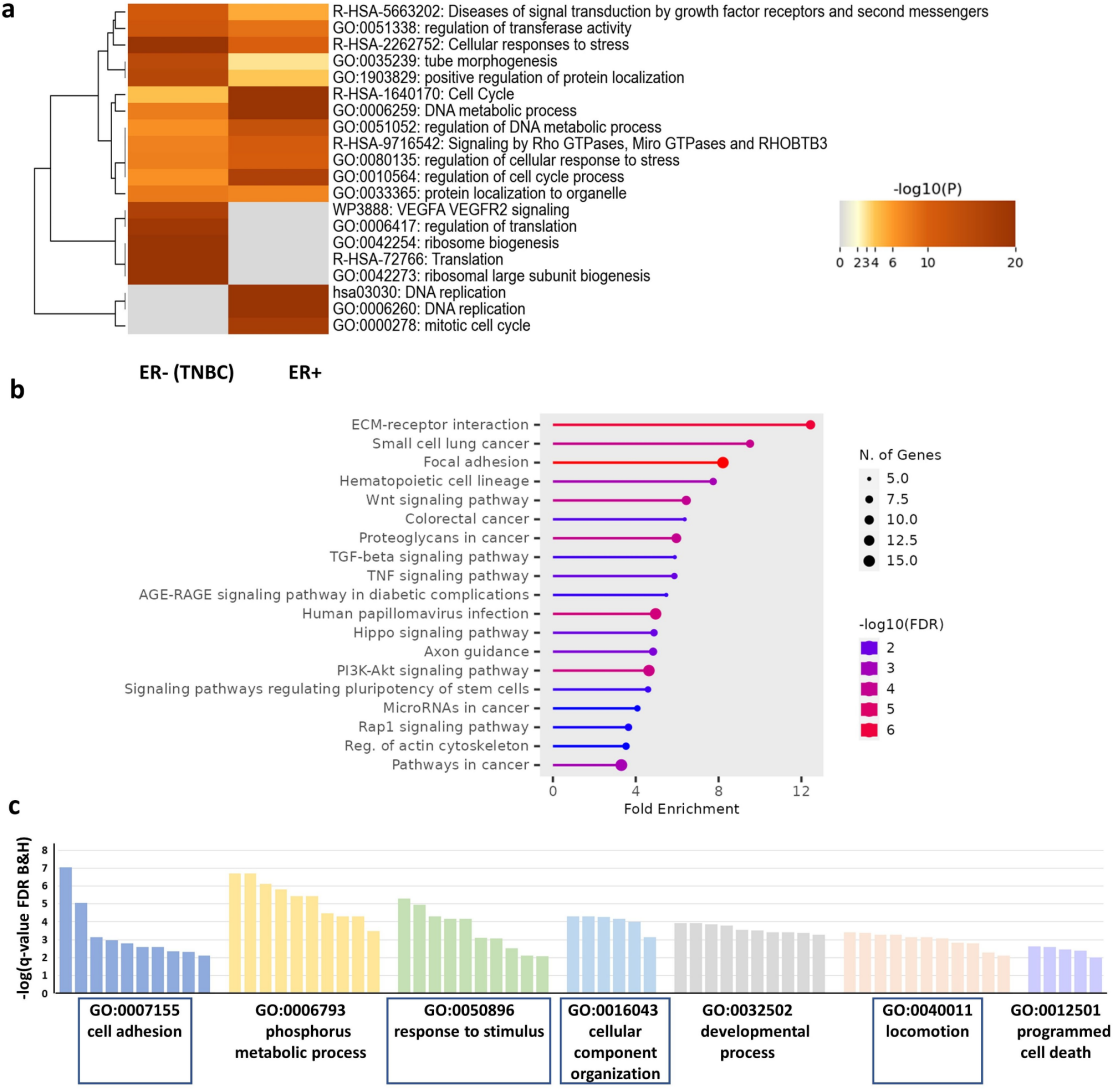
**a** Key differences in the most significantly altered biological processes and pathways in ER + and TN breast cancer cell lines following dex treatment. **b** Significant pathway enrichment of genes commonly altered by dex treatment in both TN breast cancer cell lines. **c** Significant enrichment of biological processes for genes commonly altered by dex treatment in both TN breast cancer cell lines. Details and statistical analysis of gene ontology (GO) biological process terms for differentially expressed genes following dex treatment in TN breast cancer cells are provided in Suppl. Table 2. Highlighted (framed) GO terms in panel **c** are related to cell migration, ECM, and GR signalling (see Supplementary Tables 2–3).

Following transcriptome analysis by RNA sequencing, we observed similar effects (Fig. 5C). By assessment of the transcriptional response of ER+ (ZR-75-1 & T47D) and ER- (MDA-MB231 & HS578T) cells for dex effect, we identified 999 and 2023 differentially expressed genes, respectively (Fig. 5C-D). By comparing the functions of these differences between ER + and ER- breast cancer cells we observed that the mitotic cell cycle, cellular response to stress and signalling by Rho GTPases changes were involved in different extents regarding the ER status (Fig. 6A). Only two genes, *SGK1* and *PER1* – typical GR target genes – were commonly altered between ER + and TNBC cells upon dex treatment.

Then we focused on TN breast cancer cell behaviour and the dex effect on migration behaviour. We found 228 genes differentially expressed commonly in MDA-MB231 and HS578T cells (Fig. 5D) which are implicated in ECM receptor interaction, focal adhesion, regulation of actin cytoskeleton, locomotion, TGFβ and Wnt signalling in line with the increased migration seen in vitro (Fig. 6B-C, **Suppl. Table 2**).

**Fig. 7:**
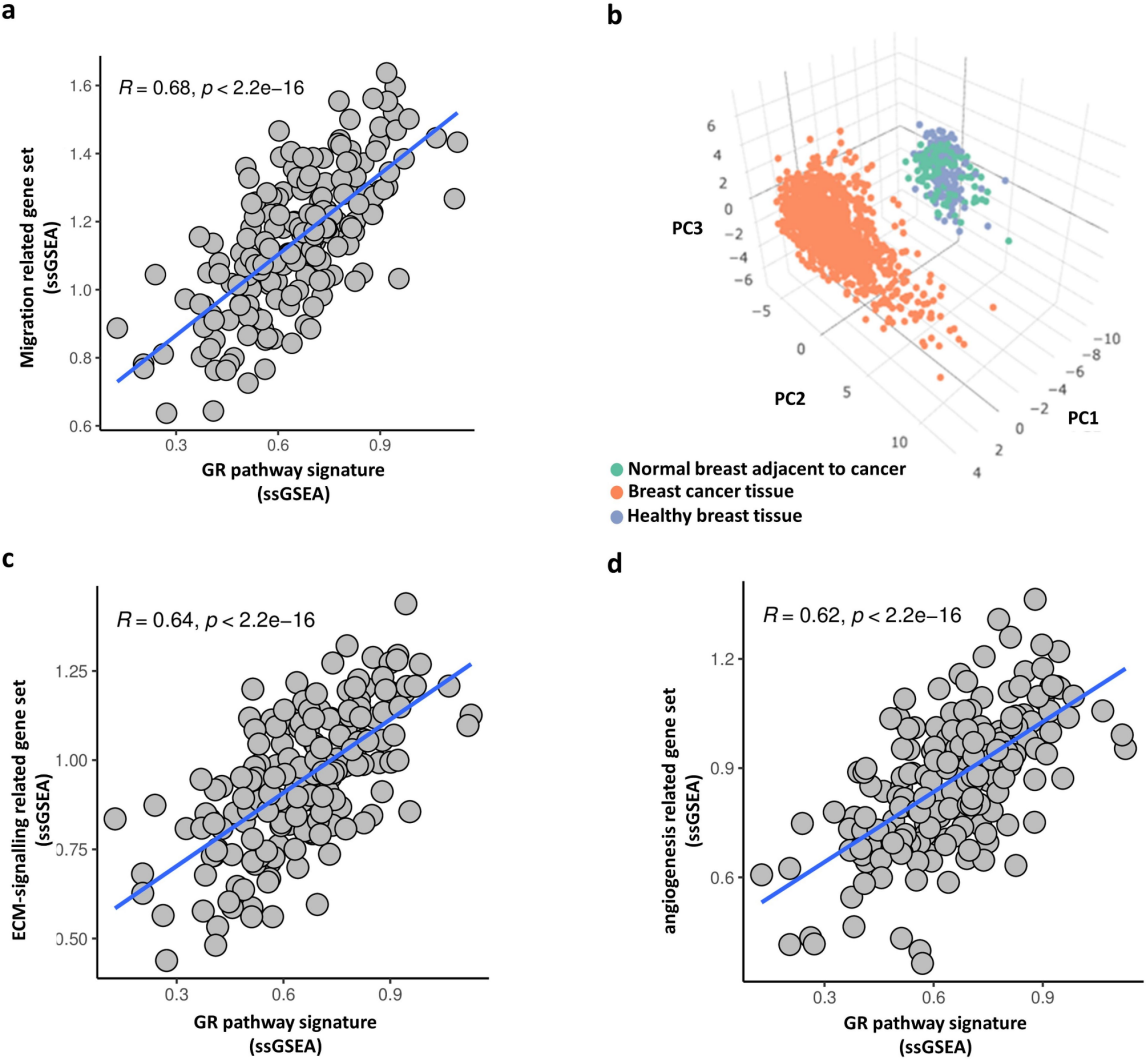
Correlations between the GR pathway (6 genes) and the migration (84 genes), ECM signalling (50 genes), and angiogenesis-related gene sets (25 genes) (for details see the Material and methods) **a** Migration-associated gene signature showed strong correlation with GR signalling pathway members’ gene signature in human breast cancer tissue specimens. **b** Migration-associated gene signature discriminated normal breast tissue from cancerous samples. **c-d** ECM and angiogenesis-related gene signature showed significant correlation with GR signalling pathway members’ gene signature.

In the next step, we cross-referenced our findings obtained in breast cancer cells with human breast cancer tissue samples. We assessed biological pathways and processes gene ontology terms enriched in dex-treated TN breast cancer cells. For this, we conducted pathway and gene ontology analyses (**Suppl. Table 3**). Given the overlap and redundancy among pathways and ontology terms, we combined the genes from significantly enriched pathways and terms, removing redundant genes to generate functionally cohesive gene sets. These included tumour progression-related (migration-related, ECM signalling-related, angiogenesis-related) and glucocorticoid receptor-related gene sets. Together, these gene sets capture the great majority of the significantly enriched pathways and ontology terms (**Suppl. Table 3**). We cross-checked these gene sets with the transcriptome of 1085 human breast cancer tissue samples. Using ssGSEA, we found significant correlations between the GR pathway and the migration-related gene set (Fig. 7A). The migratory gene set discriminated normal breast tissue from cancer samples (Fig. 7B**)**. As extracellular matrix (ECM) and vascular endothelial growth factor (VEGF) signalling are also linked to cell migration and hence metastasis development, we performed the same analysis for ECM and VEGF signalling gene sets in human samples that were altered upon dex treatment in TN cancer cells in vitro. These gene sets also strongly correlated with glucocorticoid action in the human breast cancer tissue samples, highlighting the important link between GR activation and disease progression (Fig. 7C-D).

**Fig. 8:**
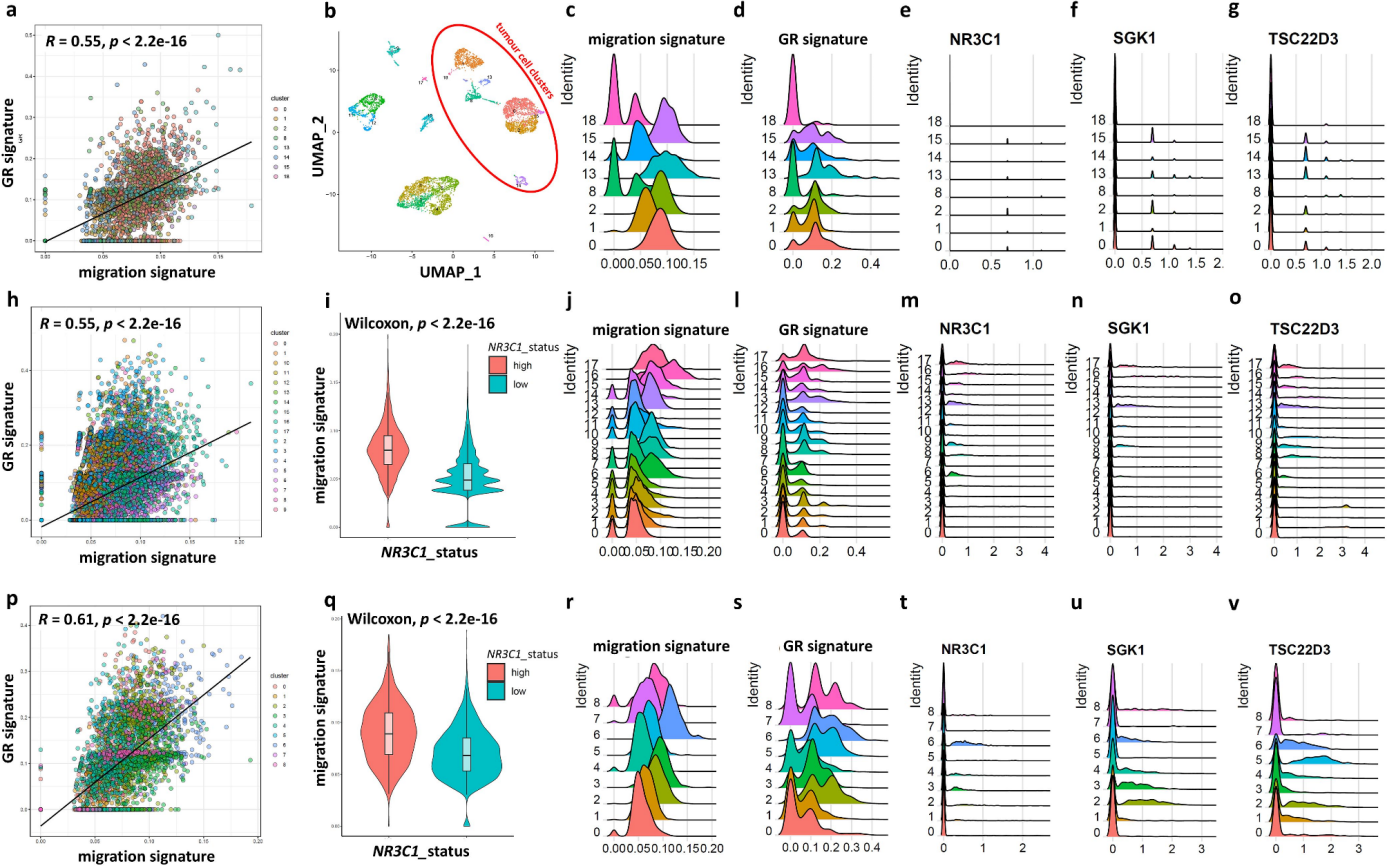
GR gene expression and GR signature in association with migration in fast and slow-migrating triple-negative single breast cancer cells. The panels depict reanalysis of single-cell sequencing data from three studies: Pal et al. 2021 (PMID: 33950524; panels **a–g**), Bassez et al. 2021 (PMID: 33958794; panels **h–o**), and Wu et al. 2021 (PMID: 34493872; panels **p–v**). **a**,** h**,** p**: Spearman correlation between GR signature and migration signature at the single-cell level across the three studies. **b**: UMAP visualization of gene expression patterns in single triple-negative breast tumour tissue cells of the study of Pal et al. 2021. generated using a dimensionality reduction algorithm. Based on cell-specific marker expression (see Supplementary Fig. 1), breast cancer cells were grouped into eight clusters (0, 1, 2, 8, 13, 14, 15, 18), highlighted by the red circle (other cell types are shown in separate clusters). These tumour cell clusters were further analysed on panel (**c-g**). Representative images of migration (**c , j , r**) and GR signature (**d , l , s**), NR3C1 (**e , m , t**), and GR target SGK1 (**f , n , u**) and TSC22D3 (**g , o , v**) gene expression in different tumour cell clusters. The migration signature between individual tumour cells with low and high GR expression were compared using the Wilcoxon test (**i , q**).

To further investigate the relationship between GR activity and cell migration, we examined GR gene expression and the GR signature in association with migration in fast and slow-migrating triple-negative, single breast cancer tumour tissue cells by in silico reanalysis of three independent single-cell sequencing studies^15–17^ (Fig. 8a-v). Using the previously identified migration-related gene set and GR signature, we observed a significant positive correlation between the GR signature and migration signature (*R* = 0.55–0.61, *p* < 2.2e-16) at the single-cell level across all three studies (Fig. 8a, h, p). Additionally, tumour cells with higher *NR3C1* expression displayed an increased migration signature (Fig. 8i, q). UMAP clustering (Fig. 8b) identified different triple-negative tumour cell clusters which were characterized by migration and GR signature and by *NR3C1*, and GR target *SGK1* and *TSC22D3* gene expression (Fig. 8c-g, j-o and **r-v**). Our analysis revealed that clusters of fast-migrating cells (with increased migration signature) exhibited significantly higher expression levels of both *NR3C1* and its targets *SGK1* and *TSC22D3*, compared to clusters of slow-migrating cells (Table 1).

**Table 1:** Gene expression of fast- and slow-migrating triple-negative breast cancer cells. Fast-migrating cells exhibit higher expression of the migratory gene set, *NR3C1* (encoding GR), and GR targets *SGK1* and *TSC22D3.*

Gene/gene set	Mean expression in fast-migrating tumour cells	Mean expression in slow-migrating tumour cells	Fold change	Wilcoxon *p*-value
Migration gene set	0.07938	0.02852	2.78	4.977e-134
*NR3C1*	0.17173	0.06729	2.55	9.160e-13
*SGK1*	0.31574	0.05253	6.01	4.436e-26
*TSC22D3*	0.30546	0.12901	2.36	7.139e-19

.

## Discussion

In the era of molecular and precision medicine, the effects and side effects of not only active treatment but also supportive therapy could be important. Therefore, the contribution of supportive medications on breast cancer progression is also of clinical importance. In recent years, studies have highlighted the potential multifaceted effect of dex on breast cancer cell growth and metastasis^3–7^, prompting us to advance our understanding of the molecular mechanisms underlying this controversial action.

Context-dependent effect of dex on cell proliferation. In breast cancer (BC), functional interaction (crosstalk) between ER and GR has recently been recognized to influence ER-mediated tumour cell proliferation^3^. In our earlier work, we observed that GR transfection of TN breast cancer cells, even in the absence of the ligand was able to increase breast cancer cell proliferation^18^. Similarly, in the current work, GR activation by the agonist dex slightly increased cell proliferation which was attenuated by GR antagonist. It should be noted that we did not include the ‘mifepristone-only’ experimental groups because previous studies have shown that mifepristone alone does not affect proliferation, cell death or migration in any of the four cell lines we tested compared with control/vehicle groups^13,19–25^. As none of these studies, using different experimental approaches, showed significant effects of mifepristone on cell proliferation or migration, we did not include ‘mifepristone-only’ treatment groups in further investigations. In addition, our study focused primarily on cell migration in TNBC cells, and our data suggest that the adverse effects of dex (a GR agonist) may be limited to triple-negative (ER and PR) cells, where PR antagonism is not relevant. However, in ER + breast cancer cells, dex treatment reduced cell growth irrespective of the presence of a GR antagonist. Indeed, ligand-activated GR was demonstrated to bind to the ER-mediated cell cycle genes’ (e.g. *CCND1*,* CDK2*, and *CDK6*) enhancer regions and repress these activated ER chromatin associations^26^. Interestingly, by performing functional studies, the authors concluded that this effect was regardless of whether the ligand is a classic GR agonist or antagonist^26^. This is in line with our results observed upon combined GR agonist and antagonist treatment in ER + cells. Accordingly, transcriptome changes upon dex treatment in ER + cells indicated altered gene expression of cell cycle regulators. On the other hand, oestrogen has the potential to trigger the dephosphorylation of the GR, which in turn could reduce the receptor’s ability to act on the specific genes involved in cell growth arrest^27^. Although there is no glucocorticoid response element identified in the promoter of ER, the indirect regulation of ER by GR has been also suggested^28^. The promoter regions of ERα are known to have numerous potential and confirmed sites for transcription factor binding^28^. A number of these sites interact indirectly with the GR. This includes the ER itself, as well as *BRCA1*, *ZEB1*, *NF-κB*, and genes related to the regulation of circadian rhythm^28,29^. These interactions between the GR and the ER have an impact on patient outcomes in ER-positive tumours, as higher expression of GR was related to a favourable prognosis, while low GR expression was associated with worse outcomes and high Ki67, p53, and CD71 expression^9^. We observed a weaker transcriptomic response in ER + compared to TN breast cancer cells. Only two genes, *SGK1* and *PER1* – typical GR target genes – were commonly altered between ER + and TNBC cells upon dex treatment indicating the strong ER-dependent effect of GR activation.

GR influence cell migration in TN breast cancer cells. Keeping in mind that TN tumours exhibit an aggressive metastatic nature, significantly compromising overall survival^30^, the investigation into dex’s effect on cell migration constitutes a foundational aspect of our work. Previously, we discovered that transfection of GR increased cell motility in ER- (TN) breast cancer cells, regardless of whether a ligand was present or absent^18^. These results suggest that since cell migration occurred regardless of the presence of ligand, the mere expression of the receptor itself could play a prognostic role. We also demonstrated that in human breast cancer samples, GR-regulated genes are implicated in cell migration^18^. Meanwhile, in the current study, we, consistent with others^13,31^, demonstrated that ligand activation also affects cell motility in both collective and single-cell migration context in TN breast cancer cells. However, this depended on the presence of ER as dex did not influence cell migration in ER + cells. Indeed, in the absence of ER, ligand-bound GR binds to the GREs of several pro-tumorigenic genes related to tumour cell survival, cell migration, and invasion corresponding with metastasis, drug resistance and hence progression in TN breast cancer^18,31,32^. Additionally, our results on ER + and TN breast cancer cells find support in other studies where TN breast cancer cells with lower migratory potential and resistance to glucocorticoid regulation exhibited decreased expression of the GR^14^. Interestingly, in TN breast cancer cells dexamethasone-induced migration phenotype was not rescued by mifepristone. This can be explained by GR-dependent but non-transcriptional mechanisms of GR activity. Our findings suggest that GR likely influences migration via both transcriptional and non-genomic actions, such as interactions with cytoplasmic signalling molecules^3^. Mifepristone primarily blocks the transcriptional activity of GR but may not effectively antagonize its non-genomic effects^33^. Nonetheless, although mifepristone is classified as a GR antagonist, studies have demonstrated its partial agonist activity as well^34^, particularly in cells with high GR expression levels. Indeed, Zhang et al. 2007^35^ demonstrated that the agonist effect of mifepristone increased proportionally with GR expression levels. In our study, we demonstrated that TNBC cell lines (MDA-MD-231 and Hs578T) expressed high levels of GR, potentially explaining the lack of mifepristone’s compensatory effect in these cells due to its partial agonist activity.

GR influences breast cancer cell migration dynamics. During time-lapse single-cell tracking, we observed significant heterogeneity in cell motility, identifying cells with both high and low migratory potential. This phenomenon is well-documented^11^, and these subpopulations with varying motility are intermingled in tumour cultures, providing the necessary plasticity for tumour cells to develop metastasis^12,36,37^. We also noticed varying migration tendencies among cultures with different seeding numbers, but this did not affect the migration-promoting effect of dex. Additionally, higher expression of *NR3C1* (encoding GR) and its targets was observed in fast-migrating cells compared to slow-migrating ones, indicating an association between GR activity and the migration characteristics of triple-negative breast cancer cells.

In terms of migration dynamics, while there is a report on dexamethasone-induced biphasic effects on TN breast cancer cell migration assessed at early (15 min) and late (6 h) phases through manual evaluation at two time points^13^, our study represents the first investigation into the impact of GR on migration dynamics through continuous time-lapse monitoring. Furthermore, our study not only examined classic wound healing but also investigated single-cell migration behaviour, providing a potentially more representative model for metastasis development. Despite this, we observed similar effects in both types of migration tests (collective and single-cell tracking); however, the transition of dex’s effect from anti-migratory to pro-migratory characteristics was observed more rapidly in single-cell tracking. This may be attributed to the absence of cell-cell adhesion compared to collective migration, though further investigation into the exact mechanisms is warranted.

Despite the growing number of studies that have undertaken preclinical functional testing, our understanding of the major driver genes and signalling pathways influenced by context-dependent GR action remains fragmentary. We attempted to elucidate the cell-type-specific action of dex by a comprehensive assessment of the transcriptome changes upon GR activation.

Interestingly, compared to the other 3 cell lines, the T47D ER + breast cancer cell line showed a weak response to dex. The main causes of glucocorticoid resistance include mutations in the *NR3C1* gene, low *NR3C1*expression, and impaired glucocorticoid signalling pathways^38^. In T47D cells, we detected very low *NR3C1* expression, which explain the weak glucocorticoid response observed via qPCR and RNA sequencing. This low *NR3C1*expression has also been validated at the protein level in this cell line by other researchers^22^. In addition, glucocorticoid signalling can be influenced by other pathways, such as PI3K. Arancibia et al.^39^showed that PI3K physically interacts with the GR through two potential binding sites and acts as a downstream effector of GR activation, indicating that PI3K’s interaction with GR could regulate some of its functions. Agarwal^40^ further demonstrated that PI3K inhibitors modify glucocorticoids’ global impact on the transcriptome and influence GR’s nuclear translocation. Additionally, Zhang et al.^41^ found that binding of dexamethasone to GR on breast cancer cells activated the PI3K signalling pathway. Although investigating PI3K’s role in glucocorticoid resistance was not the primary goal of our study, based on these previous findings, we hypothesized that the pathogenic mutation detected in the *PI3K* gene may contribute to glucocorticoid resistance in T47D cells, alongside the very low GR expression observed.

Based on our findings we propose that the dex effect on migration may be mediated through ECM, focal adhesion, Wnt, TGFβ and PI3K-Akt signalling. These pathways are connected with cancer progression and metastasis. We also demonstrated a strong correlation of GR signalling pathway members with dex-regulated migratory, ECM and angiogenesis gene set signatures in human breast cancer samples indicating the role of GR activation in these processes in human breast tumour tissues. Indeed, Obradovic et al. 2019 demonstrated that during the progression of breast cancer, stress hormones activate the GR at distant metastatic sites, leading to increased colonization and reduced survival^31^. Transcriptomics, proteomics and phospho-proteomics studies supported that the GR activation induced signalling networks and protein kinases that are implicated in breast cancer progression. Among others, *ROR1* kinase showed an increased expression in TN breast cancer cells in vitro upon dex treatment, and also in metastases compared to primary tumours in in vivo xenografts. Additionally, the downregulation of *ROR1*decreased metastasis and prolonged survival in in vivo experiments^31^. ROR1 functions in cell migration through Wnt5a, as Wnt5a induces ROR1/ROR2 hetero-oligomerization leading to the activation of Rho-GTPase RhoA and Rac1 ^42^. Wnt5a also induces ROR1 to complex with other proteins that also enhance ROR1-dependent cell migration^42^. As another mechanism, Wnt5a induces ROR1 to recruit cortactin to promote breast cancer cell migration and metastasis^43^. Wnt β-catenin-independent pathways are known to regulate cell polarity, proliferation, motility, and migration^42^. In addition, GR action also participates in these processes, which aligns with our findings.

It was also shown that GR is a required effector of TGFβ1-induced p38 MAPK signalling to advanced cancer phenotypes in TN breast cancer through regulation of cell migration^13^. In line with our previous and current findings, authors found that GR mediated TNBC migration and invasion in either the presence or absence of GR ligands^13^.

Our findings, together with those of others linking GR to tumour progression, highlight the need for further studies to draw robust conclusions about the clinical implications of dexamethasone and supportive therapy. In a recent study, we demonstrated that GR expression, assessed through immunohistochemistry in clinical breast cancer samples^44^, is associated with patient survival. If validated by further research, incorporating GR staining alongside ER and PR could provide an additional prognostic tool to identify patients who may be more susceptible to the effects of dexamethasone during chemotherapy.

Our study has several limitations. While in vitro experiments are widely used due to their simplicity, cost-effectiveness, and feasibility for drug screening and gene expression studies, they cannot fully replicate the in vivo tumour microenvironment, particularly due to the absence of cell-extracellular matrix interactions and tumour-host dynamics^45^. Conversely, in in vivo cancer models, single-cell tracking and migration dynamics are notably difficult to monitor. Additionally, we used a single dose of dexamethasone (100 nM), the most commonly used in vitro concentration, as it elicits physiologically relevant effects that approximate those seen in patients undergoing chemotherapy with dexamethasone^13,41,46,47^. However, a single-dose treatment may not fully capture the cumulative effects of repeated daily dosing, which is typical in clinical protocols^48,49^. Lastly, we were only able to investigate two ER + and two TNBC cell lines, and further studies are needed to validate these findings in additional models.

In conclusion, our findings suggest that GR activation promotes cell motility and migration specifically in TNBC cells, as observed in this study. In addition, we first assessed altered time-lapse migration dynamics in TN breast cancer cells, which may contribute to cancer progression and prognosis, highlighting the GR effect dependent on the presence of ER and dex treatment time. These findings may hold clinical relevance, as cell migration is associated with metastatic behaviour, and glucocorticoids like dexamethasone are widely used alongside chemotherapy to prevent drug-related allergic reactions and alleviate symptoms of nausea and vomiting. Thus, the impact of supportive therapy on breast cancer progression may be clinically significant, highlighting the need for further research to support the development of personalised supportive therapies for TNBC patients.

## Materials and methods

### Cell cultures and treatments

Human triple-negative (MDA-MB231 (#92020424) and HS578T (#86082104)) and oestrogen receptor-positive breast cancer cell lines T47D (#85102201) and ZR-75-1 (#87012601) were purchased from European Collection of Cell Cultures (ECACC) General Cell Collection. Cell lines were cultivated at 37 °C in a humidified atmosphere containing 5% CO2 and used until passage number 25.

HS578T cells were grown in Dulbecco’s Modified Eagle’s Medium (DMEM; #10–013-CV, Corning, Corning, NY, USA) supplemented by 10% fetal bovine serum (FBS; #P40-37500 PAN-Biotech, Germany) and 1% penicillin/streptomycin (#10378016, Thermo Fisher Scientific, Waltham, MA, USA). MDA-MB231, T47D, and ZR-75-1 breast mammary gland carcinoma cell lines were maintained in RPMI-1640 base medium (#BE12-702 F, Lonza Biosciences; Basel, Switzerland) using fetal bovine serum (FBS; #P40-37500 PAN-Biotech, Germany) in a final concentration of 10% and 1% penicillin/streptomycin (#10378016, Thermo Fisher Scientific, Waltham, MA, USA).

Three times a week, the cell culture medium was replaced with a fresh complete medium. When cells reached 90% confluence, they were detached from the bottom of the flask using 0.05% Trypsin-EDTA (#25300062, Invitrogen, Thermo Fisher Scientific, Waltham, MA, USA). Microscopic control and imaging were done by EVOS M7000 imaging system using a ×10 objective.

In experimental settings, cells were kept in steroid-free media for 48 h before plating. Steroid-free media was prepared by using charcoal-stripped FBS as previously reported^13^. Then, cells were plated on the six-well tissue culture plates (maintaining complete or steroid-free conditions). Seed numbers on the six-well plate were 50,000 and 100,000 cells/well for HS578T, T47D and MDA-MB231, ZR-5-1, respectively. On 24-well plates we seeded 20,000 and 400,000 cells/well regarding HS578T, T47D and MDA-MB231, ZR-5-1, respectively. Cells were treated with 100 nM dexamethasone and mifepristone^13^. All experiments were repeated at least three times, with one technical replicate for each sample.

Three-dimensional spheroid formation was induced by ultra-low attachment of six-well plates (07–200-601, Corning, Corning, NY, USA). Experiments were done following a 3–4-day process of spheroid induction.

### Cell proliferation ratio

Cells were seeded on 6 well plates. Cell proliferation and dead cell ratio were investigated as we previously reported^18^. Briefly, to assess cell proliferation, cell numbers were determined using 0.4% Trypan Blue staining (#15250061, Gibco, Thermo Fisher Scientific, Waltham, MA, USA). Trypan Blue staining exclusion assay is also able to identify dead cells. All experiments were repeated at least three times (biological replicates) with one to three technical replicates in each experiment. Mean and standard deviation were calculated and illustrated on bar graphs.

### Cell migration and migration dynamics analysis

To assess the effect of dex and mif on cell migration, two approaches were applied. (1) Classic wound healing assays were performed on 24-well plates as previously reported^18^, 24 h after treatment the cell monolayer was wounded using a 200-µL pipette tip and floating cells were washed with phosphate-buffered saline (PBS) (#21–040-CV, Corning, Corning, NY, USA). Photomicrographs were taken after 0, 24, and 48 or 0, 6, and 12 h depending on cell type. Images were analysed with ImageJ Software (https://imagej.nih.gov/ij/, Bethesda, MD, USA). (2) For migration dynamics analysis, time-lapse imaging was performed in every 15 min for 24 h using EVOS M7000 imaging system. Distance, accumulated distance and edge rate were analysed using Celeste 6 Image Analysis Software (Thermo Fisher Scientific, Waltham, MA, USA).

For single cell tracking, cells were seeded on 6 well plates, and time-lapse cell movies with and without treatment were recorded using EVOS M7000 imaging system using ×10 objective every 15 min. X/Y displacement, distance, accumulated distance and velocity were assessed and evaluated using Celeste 6 Image Analysis Software (Thermo Fisher Scientific, Waltham, MA, USA).

### Nucleic acid extraction

Total RNA for cell lines was isolated by the NucleoSpin RNA Mini kit for RNA purification (740955.50, Macherey-Nagel, Düren, Germany) following the manufacturer’s instructions. For total DNA extraction from cells, we used the Blood & Cell Culture DNA Kit (13323, Qiagen, Hilden, Germany) according to the manufacturer’s instructions.

### Gene expression analysis with quantitative PCR (qPCR)

A total of 750 ng of RNA was transcribed by the ProtoScript^®^ II Reverse Transcriptase (M0368L, New England Biolabs, Ipswich, MA, USA). Gene expression was measured by reverse transcription–quantitative polymerase chain reaction (RT-qPCR) using TaqMan™ Fast Universal PCR Master Mix (2X) no AmpErase™ UNG (4367846, Thermo Fisher Scientific, Waltham, MA, USA) and TaqMan™ Gene Expression Assays (FAM) (4331182, Thermo Fisher Scientific, Waltham, MA, USA), including the following assays NR3C1: Hs00353740_m1; TSC22D3: Hs00608272_m1; SGK1: Hs00178612_m1. Glyceraldehyde 3-phosphate dehydrogenase (Hs02786624_g1, Thermo Fisher Scientific, Waltham, MA, USA) was used as an endogenous control. Quantitative PCRs were run on QuantStudio 5 Real-Time PCR System (Thermo Fisher Scientific, Waltham, MA, USA). Gene expression was calculated using the formula for fold change [fold change = 2^−ΔΔCt^].

### Transcriptome sequencing

The libraries for Illumina sequencing were prepared using xGen RNA Lib Prep Kit (Integrated DNA Technologies, Iowa, USA). Briefly, mRNA was isolated from 400 ng of total RNA using Poly(A) RNA Selection Kit V1.5 (Lexogen, Vienna, Austria). Thereafter, the mRNA was fragmented and reverse-transcribed using random priming, then the cDNA was tailed and adapter-ligated. Finally, the libraries were amplified according to the manufacturer’s instructions. The quality of the libraries was checked on Agilent 4200 TapeSation System using D1000 Screen Tape (Agilent Technologies, Palo Alto, CA, USA), and the quantity was measured on Qubit 3.0 (Thermo Fisher Scientific, Waltham, MA, USA). Illumina sequencing was performed on a NovaSeq 6000 instrument (Illumina, San Diego, CA, USA) with a 2 × 151 cycles run configuration.

### Bioinformatic analysis

Sequenced RNA-seq data were mapped to the human genome (hg38) obtained from Gencode using the v44 annotation. Reads were mapped using the *STAR*aligner^50^version 2.7.11a, applying previously published benchmarked settings^51^. Raw read quantification was obtained by *FeatureCounts*version 2.0.2^52^. Differential expression analysis was performed using *DESeq2*^53^ version 1.38.3, while TMM-FPKM normalized expression was calculated using *edgeR*^54^ version 3.40.2 in R (v4.2.1). Expression heatmap was plotted using the *ComplexHeatmap*^55^ package (version 2.14.0) in R.

Gene set enrichment analysis for pathways and gene ontology categories was done using Metascape, ShinyGO and ToppGene Suite algorithms^56–58^. Statistically enriched terms (can be GO/KEGG terms and canonical pathways) were identified and accumulative hypergeometric p-values and enrichment factors were calculated and used for filtering. The remaining significant terms were then hierarchically clustered into a tree based on Kappa-statistical similarities among their gene memberships. Then 0.3 kappa score was applied as the threshold to cast the tree into term clusters. The term with the best p-value within each cluster was selected as its representative term and it was displayed in a dendrogram. The heatmap cells are coloured by their p-values, white cells indicate the lack of enrichment for that term in the corresponding gene list.

As a validation set, gene expression signature was extracted from 1085 breast tumours, 112 normal breast tissue specimens through The Cancer Genome Atlas (TCGA) integrated cohort^59^, and 179 normal breast tissues through GTEx Portal (https://gtexportal.org/home/, accessed on 24 April, 2024). ssGSEA using the *ssgsea* function of the *corto*(version 1.2.2)^60^ package in R (version 4.2.1) was used for gene signature correlations to prevent results from being skewed by highly abundant genes.

Single-cell RNA-seq data of TNBC samples proficient for *BRCA1* were obtained from *Pal et al.*^15^ through the NCBI GEO database (accession numbers: GSM4909281, GSM4909282, GSM4909283 and GSM4909284), from *Bassez et al. 2021*^16^. and *Wu et al. 2021*^17^. Altogether, single cell sequencing data of 31 TNBC patients were reanalysed. Expression data was loaded with the Seurat^61^package (version 4.4.0) in R (version 4.2.1), downsampling each patient to 2k cells. Cells were integrated and normalized (SCTransform), followed by statistical analysis. Gene set enrichment for migration genes was calculated using the escape^62^ package (version 1.8.0), using the „UCell” method with default settings. All analyses and statistics were calculated on 45,114 cells (average 1455 cell/patient).

### Genetic analysis of breast cancer cells

Multigene panel testing was performed using the TruSight Hereditary Cancer Panel (#20029551, Illumina, San Diego, CA, USA) library preparation kit, including classic tumour suppressors and oncogenes. Next-generation sequencing (NGS) was run on Illumina MiSeq instrument with MiSeq Reagent Kit v3 (600 cycles) (#MS-102–2002, Illumina, San Diego, CA, USA). Data analysis was performed using the Illumina Dragen Enrichment pipeline (Dragen version 4.0.3, Illumina) to uncover sequence variants, copy number alterations and potential structural variants. GRCh37 genome build and NCBI MANE Select transcripts were used as reference sequences. Variants were classified following the ACMG guideline for variant interpretation using the Franklin Genoox database (https://franklin.genoox.com/clinical-db/home).

### Statistical methods

For the comparison of multiple groups, we used analysis of variance (ANOVA) to assess statistical significance between the groups. Post-hoc comparisons were performed using either Dunnett’s test, to compare each mean with a control mean, or Tukey’s test, to compare every mean with every other mean. When comparing two groups, either an unpaired t-test or Mann-Whitney test was used, depending on the distribution of the data. Comparing gene expression of fast and slow migrating cells and cells with high and low *NR3C1* expression from the single cell sequencing experiment, Wilcoxon rank sum test was applied. Spearman correlation was used to investigate the association between GR and migration signature. A p-value of < 0.05 was considered statistically significant. To examine the correlation between *NR3C1* and the expression of other genes in the RNA sequencing data, Pearson’s correlation coefficient was calculated.

## Electronic supplementary material

Below is the link to the electronic supplementary material.

Supplementary Material 1

Supplementary Material 2

Supplementary Material 3

## Data Availability

All data are presented in the manuscript; public databases from which validation data were obtained are indicated in the Methods section. Transcriptomic data will be deposited in a publicly available database upon acceptance.
