# Supplementary material for: The impact of glucocorticoid receptor transactivation on context-dependent cell migration dynamics
Source: Sci Rep. 2025 Feb 4;15:4163. doi: 10.1038/s41598-025-88666-1 (PMC11794636; doi:10.1038/s41598-025-88666-1)
Supplement: Supplementary file 2 — Supplementary Material 2 [file 41598_2025_88666_MOESM2_ESM.docx]

**The Impact of Glucocorticoid Receptor Transactivation on Context-dependent Cell Migration Dynamics** *by Szonja Polett Pósa, Éva Saskői, Lili Bársony, Lőrinc Pongor, Fanni Fekete, János Papp, Anikó Bozsik, Attila Patócs and Henriett Butz*

**Supplementary Table 1.** Genetic variants of tumour suppressors and oncogenes identified in MDA-MB231, HS578T, ZR-75-1 and T47D cells

Abbreviations: ACMG: American College of Medical Genetics and Genomics; HGVS: Human Genome Variation Society VAF: variant allele frequency; e:exon; i: intron; P/LP: pathogenic/likely pathogenic; VUS: variant of uncertain significance

| **Gene** | **Variant name (according to HGVS)** | **Variant Type**  **(affected exon #/ all exon #)** | **ACMG classification (according to Franklin genoox database)** | **MDA-MB231**  **(read#\|VAF)** | **HS578T**  **(read#\|VAF)** | **ZR-75-1**  **(read#\|VAF)** | **T47D**  **(read#\|VAF)** |
| --- | --- | --- | --- | --- | --- | --- | --- |
| *MEN1* | NM_001370259.2:c.1368delC, p.(Ile457Ter) | stop_gained  (e10/10) | LP | - | - | 1083\|1.00 | - |
| *MSH3* | NM_002439.5:c.2686G>A,  p.(Gly896Arg) | missense  (e20/24) | LP | 165\|0.42 | - | - | - |
| *NF1* | NM_001042492.3:c.8233G>A,  p.(Gly2745Arg) | missense  (e57/58) | VUS | - | 270\|0.53 | - | - |
| *NF1* | NM_001042492.3:c.1398_1399insC,  p.(Thr467HisfsTer3) | frameshift  (e13/58) | LP | 92\|1.00 | - | - | - |
| *NF2* | NM_000268.4:c.691G>T,  p.(Glu231Ter) | stop_gained  (e8/16) | LP | 136\|1.00 | - | - | - |
| *PDGFRA* | NM_006206.6:c.515_516delACinsT,  p.(Tyr172LeufsTer13) | frameshift  (e4/23) | VUS | 268\|0.33 | - | - | - |
| *PIK3CA* | NM_006218.4:c.3140A>G,  p.(His1047Arg) | missense  (e21/21) | P | - | - | - | 948\|0.85 |
| *POT1* | NM_015450.3:c.702+8_702+9delATinsTG,  p.(?) | splice_region  (i9/18) | VUS- | - | - | 101\|0.24 | - |
| *PTEN* | NM_000314.8:c.323T>G,  p.(Leu108Arg) | missense  (e5/9) | LP | - | - | 265\|1.00 | - |
| *PTEN* | NM_000314.8:c.802-4_802-3delTT,  p.(?) | splice_region  (i7/8) | VUS- | - | 61\|0.26 | - | - |
| *RAD51D* | NM_002878.4:c.*1C>A,  p.(?) | 3'UTR  (e10/10) | VUS- | 179\|1.00 | - | - | - |
| *RET* | NM_020975.6:c.265C>A,  p.(Gln89Lys) | missense  (e2/20) | VUS | - | - | - | 1752\|0.23 |
| *TP53* | NM_000546.6:c.469G>T,  p.(Val157Phe) | missense  (e5/11) | P | - | 1717\|1.00 | - | - |
| *TP53* | NM_000546.6:c.839G>A,  p.(Arg280Lys) | missense  (e8/11) | P | 383\|1.00 | - | - | - |
| *TP53* | NM_000546.6:c.580C>T,  p.(Leu194Phe) | missense  (e6/11) | P | - | - | - | 761\|1.00 |

**Supplementary Table 2.** Hierarchical gene ontology biological process terms of differentially expressed genes upon dex treatment in TN breast cancer cells

GO-BP: gene ontology biological process; FDR B&H: false discovery rate correction by Benjamini-Hochberg procedure

| **GO-BP ID** | **GO-BP term** | **q-value FDR B&H** |  |
| --- | --- | --- | --- |
| **GO:0007155 - cell adhesion** | | | |
| GO:0007155 | cell adhesion | 9.28E-08 |  |
| GO:0031589 | cell-substrate adhesion | 8.77E-06 |  |
| GO:0007160 | cell-matrix adhesion | 7.23E-04 |  |
| GO:0098609 | cell-cell adhesion | 1.02E-03 |  |
| GO:0034446 | substrate adhesion-dependent cell spreading | 1.63E-03 |  |
| GO:0033627 | cell adhesion mediated by integrin | 2.61E-03 |  |
| GO:0045785 | positive regulation of cell adhesion | 2.63E-03 |  |
| GO:0010810 | regulation of cell-substrate adhesion | 4.31E-03 |  |
| GO:0034113 | heterotypic cell-cell adhesion | 4.93E-03 |  |
| GO:0010811 | positive regulation of cell-substrate adhesion | 7.69E-03 |  |
| **GO:0006793 - phosphorus metabolic process** | | | |
| GO:0010562 | positive regulation of phosphorus metabolic process | 1.99E-07 |  |
| GO:0045937 | positive regulation of phosphate metabolic process | 1.99E-07 |  |
| GO:0042327 | positive regulation of phosphorylation | 7.44E-07 |  |
| GO:0042325 | regulation of phosphorylation | 1.53E-06 |  |
| GO:0019220 | regulation of phosphate metabolic process | 3.75E-06 |  |
| GO:0051174 | regulation of phosphorus metabolic process | 3.75E-06 |  |
| GO:0001934 | positive regulation of protein phosphorylation | 3.27E-05 |  |
| GO:0006468 | protein phosphorylation | 4.90E-05 |  |
| GO:0001932 | regulation of protein phosphorylation | 4.90E-05 |  |
| GO:0001822 | kidney development | 3.18E-04 |  |
| **GO:0050896 - response to stimulus** | | | |
| GO:0007167 | enzyme-linked receptor protein signaling pathway | 5.03E-06 |  |
| GO:0009611 | response to wounding | 1.12E-05 |  |
| GO:0070848 | response to growth factor | 4.90E-05 |  |
| GO:0071363 | cellular response to growth factor stimulus | 6.82E-05 |  |
| GO:0030155 | regulation of cell adhesion | 6.82E-05 |  |
| GO:0007169 | transmembrane receptor protein tyrosine kinase signaling pathway | 7.69E-04 |  |
| GO:0071495 | cellular response to endogenous stimulus | 8.12E-04 |  |
| GO:0042060 | wound healing | 3.05E-03 |  |
| GO:0071559 | response to transforming growth factor beta | 7.68E-03 |  |
| GO:0034097 | response to cytokine | 8.55E-03 |  |
| **GO:0016043 - cellular component organization** | | | |
| GO:0030198 | extracellular matrix organization | 4.90E-05 |  |
| GO:0043062 | extracellular structure organization | 4.90E-05 |  |
| GO:0045229 | external encapsulating structure organization | 5.15E-05 |  |
| GO:0007044 | cell-substrate junction assembly | 6.55E-05 |  |
| GO:0150115 | cell-substrate junction organization | 9.91E-05 |  |
| GO:0034330 | cell junction organization | 7.23E-04 |  |
| **GO:0032502 - developmental process** | | | |
| GO:0007423 | sensory organ development | 1.15E-04 |  |
| GO:0072073 | kidney epithelium development | 1.16E-04 |  |
| GO:0043010 | camera-type eye development | 1.39E-04 |  |
| GO:0048729 | tissue morphogenesis | 1.54E-04 |  |
| GO:0042063 | gliogenesis | 2.88E-04 |  |
| GO:0072006 | nephron development | 2.98E-04 |  |
| GO:0072009 | nephron epithelium development | 3.84E-04 |  |
| GO:0009887 | animal organ morphogenesis | 3.84E-04 |  |
| GO:0072001 | renal system development | 4.07E-04 |  |
| GO:0048880 | sensory system development | 5.43E-04 |  |
| **GO:0040011 - locomotion** | | | |
| GO:0006935 | chemotaxis | 3.96E-04 |  |
| GO:0042330 | taxis | 4.00E-04 |  |
| GO:0040017 | positive regulation of locomotion | 5.33E-04 |  |
| GO:0030335 | positive regulation of cell migration | 5.43E-04 |  |
| GO:0040011 | locomotion | 7.23E-04 |  |
| GO:0030334 | regulation of cell migration | 7.28E-04 |  |
| GO:2000147 | positive regulation of cell motility | 8.12E-04 |  |
| GO:0040012 | regulation of locomotion | 1.46E-03 |  |
| GO:2000145 | regulation of cell motility | 1.54E-03 |  |
| GO:0050921 | positive regulation of chemotaxis | 4.94E-03 |  |
| GO:0050920 | regulation of chemotaxis | 7.68E-03 |  |
| **GO:0012501 - programmed cell death** | | | |
| GO:0043066 | negative regulation of apoptotic process | 2.28E-03 |  |
| GO:0042981 | regulation of apoptotic process | 2.46E-03 |  |
| GO:0043069 | negative regulation of programmed cell death | 3.36E-03 |  |
| GO:0043067 | regulation of programmed cell death | 4.00E-03 |  |
| GO:0043523 | regulation of neuron apoptotic process | 9.60E-03 |  |
