# Supplementary figures and images for: The impact of glucocorticoid receptor transactivation on context-dependent cell migration dynamics
Source: Sci Rep. 2025 Feb 4;15:4163. doi: 10.1038/s41598-025-88666-1 (PMC11794636; doi:10.1038/s41598-025-88666-1)

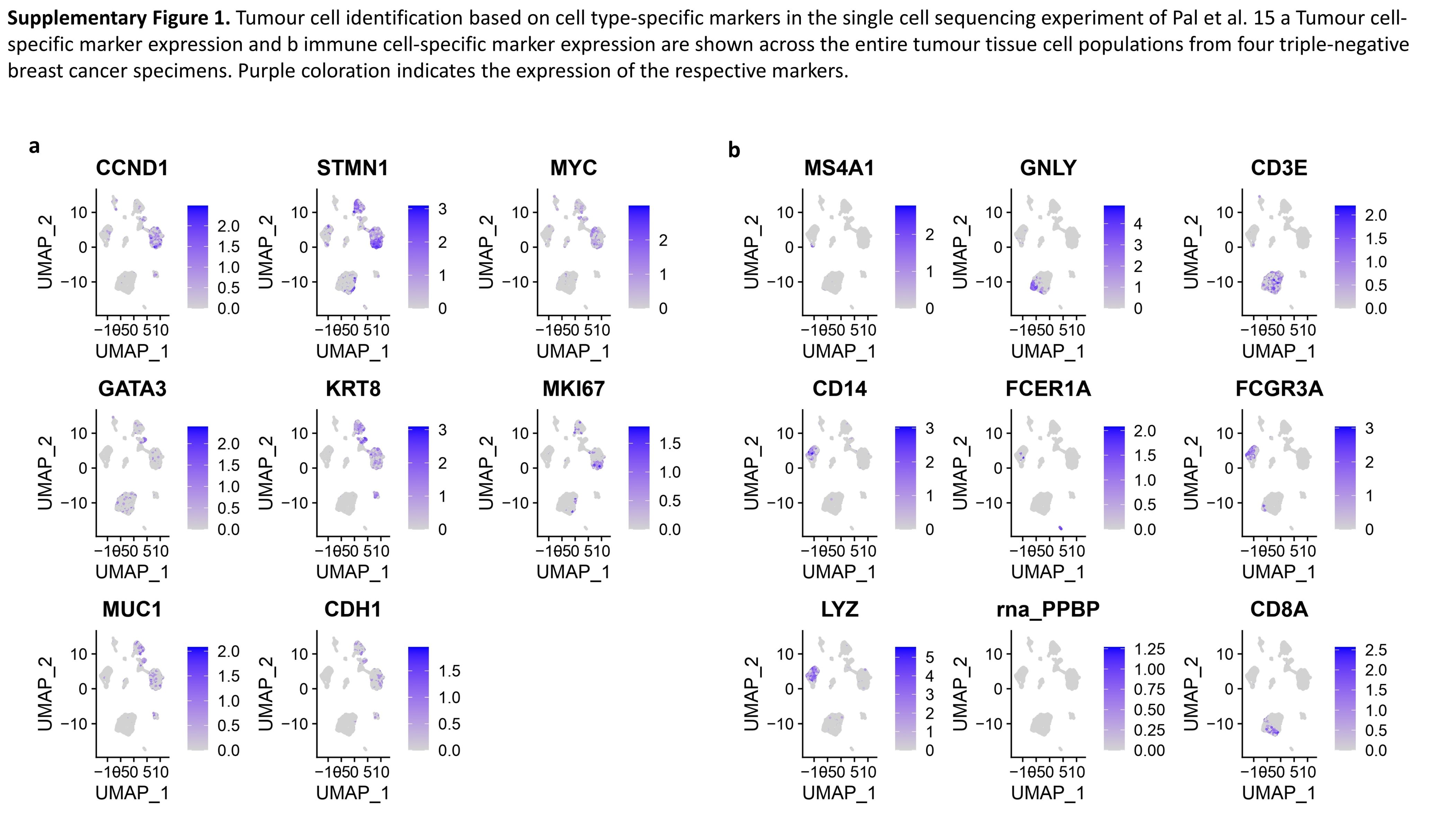

Supplement: Supplementary file 3 — Supplementary Material 3 [file 41598_2025_88666_MOESM3_ESM.jpg]
